# Side Effects of First-Line Anti-tubercular Therapy (ATT): Does an Alternative Regimen Exist?

**DOI:** 10.7759/cureus.100391

**Published:** 2025-12-30

**Authors:** Gauri Goswami, Pradeep Nirala, Rajeev Tandon, Pulkit Kalra, Mohammed Tariq, Lalit Singh

**Affiliations:** 1 Respiratory Medicine, Royal Oldham Hospital, Manchester, GBR; 2 Respiratory Medicine, Teerthanker Mahaveer Medical College and Research Centre, Moradabad, IND; 3 Respiratory Medicine, Shri Ram Murti Smarak Institute of Medical Sciences, Bareilly, IND; 4 Orthopaedics, Royal Oldham Hospital, Manchester, GBR; 5 Respiratory Medicine and Critical Care, Narayan Swaroop Hospital, Prayagraj, IND; 6 Respiratory Medicine and Critical Care, Shri Ram Murti Smarak Institute of Medical Sciences, Bareilly, IND

**Keywords:** drug-induced liver injury (dili), drug related adverse effects, gi intolerance, hyperurecemia, non standardized antitubercular regimen

## Abstract

Setting

Standard anti-tubercular therapy (ATT) typically involves a regimen of 2 months of HRZE intensive phase, followed by 4 months of HRE continuation phase - 2HRZE/4HRE (H-isoniazid, R-rifampicin, Z-pyrazinamide, E-ethambutol). However, adverse effects and comorbidities often necessitate alternative non-standardized regimens.

Objective

The aim of the study was to identify and evaluate the use, rationale, and outcome of non-standardized ATT regimens in patients with drug-sensitive tuberculosis.

Method

Our prospective observational study included 148 patients at a tertiary care hospital who were prescribed non-standard ATT due to various adverse effects and associated medical comorbidities on or after presentation. Patients were followed up through the intensive phase (two months) of the Directly Observed Treatment, Short-course (DOTS) regimen only, and their progress was assessed before the onset of the continuation phase.

Results

The most common reasons for non-standardized ATT were drug-induced liver injury (DILI) - 53.4%(79), and gastrointestinal (GI) intolerance - 21.6% (32). HRE was the most frequently used alternative regimen prescribed to 35.1% (52) of patients. At the end of the intensive phase, 76% (108) of patients reported clinical improvement, and 60.1%(89) showed radiological resolution. Three patients passed away during the course of the study, while four were lost to follow-up. No adverse effects were observed with modified regimens.

Conclusion

Non-standardized ATT regimens are often necessary due to adverse drug reactions, particularly hepatotoxicity. Most patients experienced symptomatic and radiological improvement on a non-standardized ATT regimen.

## Introduction

India accounts for nearly 20% of the global tuberculosis (TB) burden. The National Tuberculosis Elimination Program (NTEP), using DOTS, has significantly improved TB detection (70%) and cure rates (85%). India remains one of the top three TB-burdened countries, with an estimated 26.9 lakh new cases (WHO) [1]. The standard regimen of *2HRZE/4HRE* (daily dose regimen) started in 2016 and is preferred due to its effectiveness, simplicity, and resource efficiency. It reduces prescription errors, improves logistics, and allows better patient management [2]. In patients with liver/kidney disease, pregnancy, diabetes, hypersensitivity, GI intolerance, or specific physician judgment, the standard treatment regimen for treatment of TB requires modification.

Isoniazid, rifampicin, and pyrazinamide are hepatotoxic drugs [3]. Patients with chronic liver disease should avoid pyrazinamide. For milder dysfunction, modified regimens using one or two hepatotoxic drugs are suggested. In acute hepatitis, therapy may be delayed or started with S (streptomycin) E ± quinolones [2,4]. Nausea and discomfort can be mitigated by taking TB meds at night or with light snacks. Antacids may be used, avoiding those containing calcium/zinc near fluoroquinolone dosing [5]. Cutaneous reactions are common, with ethambutol being the most implicated. On recovery, drugs are reintroduced sequentially to identify the culprit. Severe reactions (e.g., Stevens-Johnson syndrome) require permanent discontinuation of the offending drug [6]. Pyrazinamide leads to elevated serum uric acid levels; however, most of these patients are managed symptomatically. Drug withdrawal is only considered for persistent, symptomatic cases. Allopurinol is not routinely recommended for asymptomatic cases [7]. Isoniazid, rifampicin, and pyrazinamide are generally safe in patients with kidney function compromise; however, ethambutol requires dose frequency adjustment. Drugs are usually given post-hemodialysis with regular close monitoring of serum drug levels and renal function [8]. All first-line drugs are safe in pregnancy, with pyrazinamide strongly recommended for patients with HIV or extrapulmonary TB [9]. Diabetic patients may face an increased incidence of drug toxicity, gastroparesis, and renal dysfunction. Monitoring renal function, managing neuropathy, and regular drug level monitoring are essential.

Although the Revised National Tuberculosis Control Programme (RNTCP) has involved private practitioners across the nation through various Public-Private Mix schemes for training, variation in prescriptions and lack of standardization contribute to the rising incidence of multi-drug resistant tuberculosis (MDR-TB) cases [10]. Through this study, we aim to identify the various non-standardized regimens in prevalence and observe their effectiveness.

## Materials and methods

Objectives

The objectives of this study were: (1) to identify various prescribed non-standardized ATT regimens, (2) to determine the reasons for prescription of non-standardized ATT regimens, and (3) to evaluate acceptance, tolerance, and patient outcomes at the end of the intensive phase.

Methods

This prospective observational study included 148 patients as the sample. These patients were diagnosed with drug-sensitive pulmonary or extrapulmonary TB and were managed on a non-standardized ATT regimen at our tertiary care hospital. Their treatment was started in Pulmonology outpatient clinics by experienced chest physicians. All these patients experienced adverse effects within one month after initiation of the standardized ATT regimen. Attempts were made to sequentially add one drug at a time (usually per week) from a standardized regimen alongside non-standardized regimen medication. Patients restarted on standardized ATT after successful rechallenge were excluded from this study. All patients were followed up for two months of the intensive phase.

Formula used for calculating the sample size: n = $\frac{Z_{\alpha/2}^{2} \times P \times (100 - P)}{E^{2}}$, where Z^2^_ɑ/__2 _is the standard normal variate at the desired level of confidence interval, P is the prevalence in the population based on a previous study or a pilot study, and E is the absolute error.

Here, ​​​​​​​Z^2^_ɑ/2_= 1.96 at 95% confidence interval, P = 15%, and E = 6%.

On substituting the above values in the formula, n = $\frac{1.96 \times 1.96 \times 15(100 - 15)}{6 \times 6}$ = 136.

Based on published evidence reporting approximately 9% incidence of major adverse drug reactions and considering additional regimen modifications due to associated comorbidities during the intensive phase, the expected proportion of non-standard ATT use was assumed to be 15%. Using 95% confidence level and 6% absolute precision, the minimum sample size was calculated as 136.

After considering 5% loss to follow-up, the final sample size was 143. 

All patients of tuberculosis, outpatient and inpatient, confirmed as drug-sensitive tuberculosis by polymerase chain reaction (PCR) testing, or who did not fall in the case definition of presumptive drug-resistant tuberculosis, were included in this study. Patients with confirmed/presumptive drug-resistant tuberculosis, previously treated patients, pediatric patients, and those diagnosed with HIV constituted the exclusion criteria of the study.

Patients were categorized based on the reason for deviation from standard therapy. All patients had investigations, including complete blood count, liver profile, renal profile, and viral markers, which served as the baseline. Diagnosis was confirmed using microbiology or clinically, as per the national guidelines.

Data was collected using a set proforma, and descriptive study analysis was done using SPSS Statistics (IBM Corp., Armonk, USA).

Ethical clearance was secured after approval from the Institutional Review Board of the Shri Ram Murti Smarak Institute of Medical Sciences (approval number SRMSIMS/ECC/PG-20/2020-21/041). Written informed consent was obtained from all patients before they were enrolled in the study.

## Results

All patients in our study were switched to non-standardized ATT within one month of being diagnosed with TB. In our study, males constituted 56.8% (84), while 43.2% (64) were females. Patients in our study ranged from 18 years to 90 years, with the majority, 34.5% (51), being between 41 and 60 years. 25% (37) of the total population being prescribed non-standardized ATT were more than 60 years of age. After starting standardized ATT, serology was checked regularly, and within the first month, 53.4% (79) had a deranged liver profile, 9.5% (14) had impaired renal function, while 7.4% (11) had elevated serum uric acid. Seven patients were hepatitis B or C positive before initiating treatment, with six of them developing drug-induced liver injury DILI during the first month of treatment.

The patients were profiled in accordance with the reason they were started on non-standardized ATT therapy other than HRZE. The various non-standardized regimens that were prescribed in our study were HRE in 35.1% (52), SE with levofloxacin (Levo) in 16.9%(25), E-Levo in 15.5%(23), RE-Levo in 2.7% (4), and others like amikacin (Am)-E-Levo, SHRE, SE-Levo, HRZ, RH-E-Levo-Lzd (linezolid), and RH (Table 1). Out of the total patients (148), the most common reason for conversion to a non-standardized regimen was DILI in 53.4%(79), second being GI Intolerance in 21.6 %(32), followed by Renal Impairment(14) and hyperuricemia(11). Pregnancy, cutaneous toxicities, and doctor preference were infrequent reasons for prescribing Non- standardized ATT.

**Table 1:** Alternative ATT regimens started due to various adverse effects (n=number of patients) DILI: drug-induced liver injury; ATT:  anti-tubercular therapy; E-Levo: ethambutol plus levofloxacin; HE-Levo: isoniazid, ethambutol, and levofloxacin; HRE: isoniazid, rifampicin, and ethambutol; RE-Levo: rifampicin, ethambutol, and levofloxacin; SE-Levo: streptomycin and levofloxacin.

Adverse effect experienced		Alternate regimen started												Total	
		E-Levo		HE-Levo		HRE		RE-Levo		SE-Levo		Others			
		n	%	n	%	n	%	n	%	n	%	n	%	n	%
Reason for Non-standardised Regimens	DILI	19	82.60%	11	73.30%	20	38.50%	0	0.00%	15	60.00%	14	48.30%	79	53.40%
	GI Intolerance	4	17.40%	2	13.30%	16	30.80%	1	25.00%	6	24.00%	3	10.30%	32	21.60%
																Renal Impairment	0	0.00%	0	0.00%	7	13.50%	0	0.00%	1	4.00%	6	20.70%	14	9.50%
	Hyperuricemia	0	0.00%	0	0.00%	7	13.50%	1	25.00%	0	0.00%	3	10.30%	11	7.40%															
	Intolerance	0	0.00%	1	6.70%	2	3.80%	0	0.00%	2	8.00%	0	0.00%	5	3.40%
	Skin Rashes	0	0.00%	1	6.70%	0	0.00%	1	25.00%	1	4.00%	0	0.00%	3	2.00%
																Vision Impairment	0	0.00%	0	0.00%	0	0.00%	1	25.00%	0	0.00%	1	3.40%	2	1.40%
	Pregnancy	0	0.00%	0	0.00%	0	0.00%	0	0.00%	0	0.00%	1	3.40%	1	0.70%															
	Dr Preference	0	0.00%	0	0.00%	0	0.00%	0	0.00%	0	0.00%	1	3.40%	1	0.70%
																Total		23	100.00%	15	100.00%	52	100.00%	4	100.00%	25	100.00%	29	100.00%	148	100.00%

**Figure 1:**
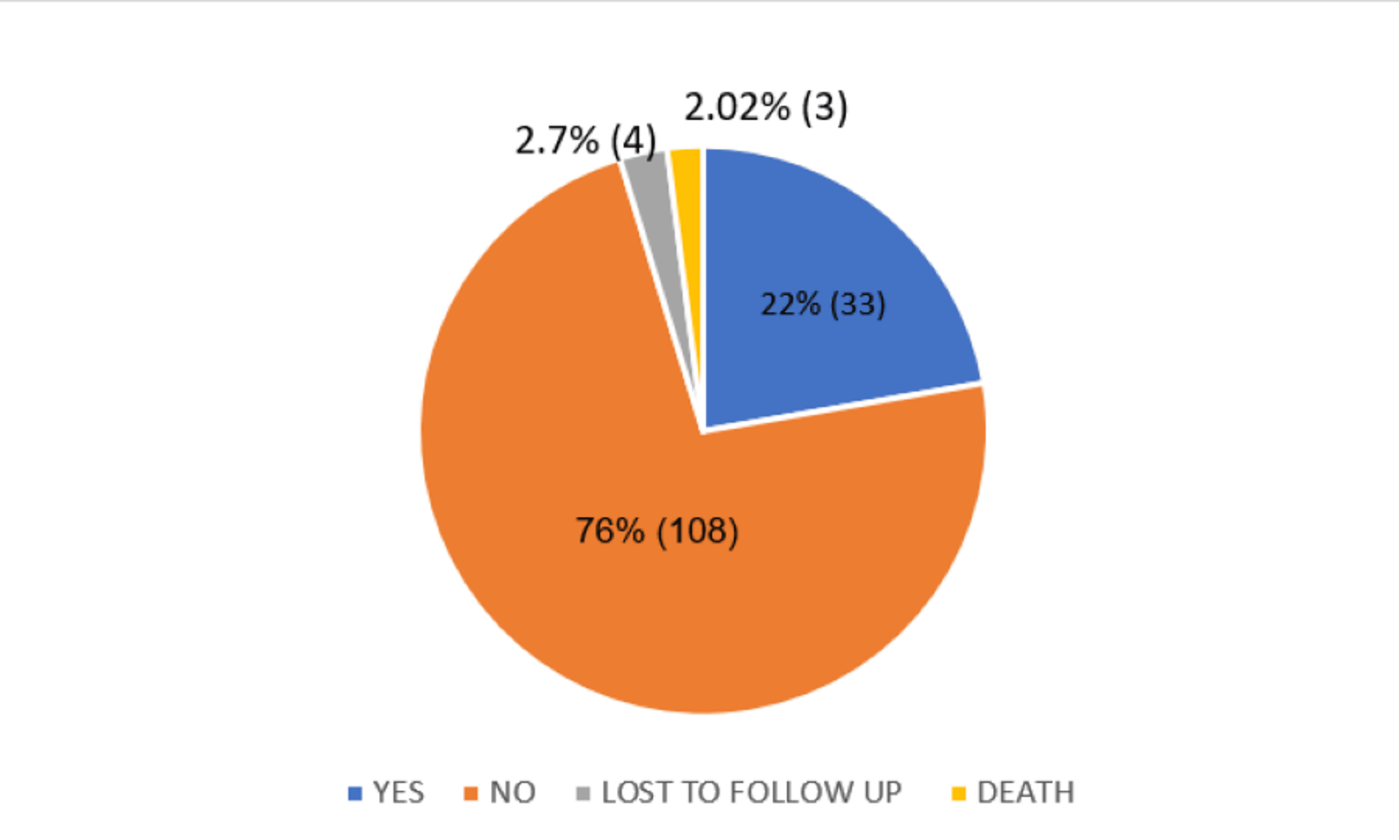
Patients’ clinical symptoms after managing on alternative regimens at the end of two months of follow up (frequencies and percentages)

Among the 141 patients completing the study, 76% (108) patients had clinical improvement, while 22% (33) patients had partial or no relief in symptoms like cough, breathlessness, and back pain (Pott’s spine) (Figure 1).

**Figure 2:**
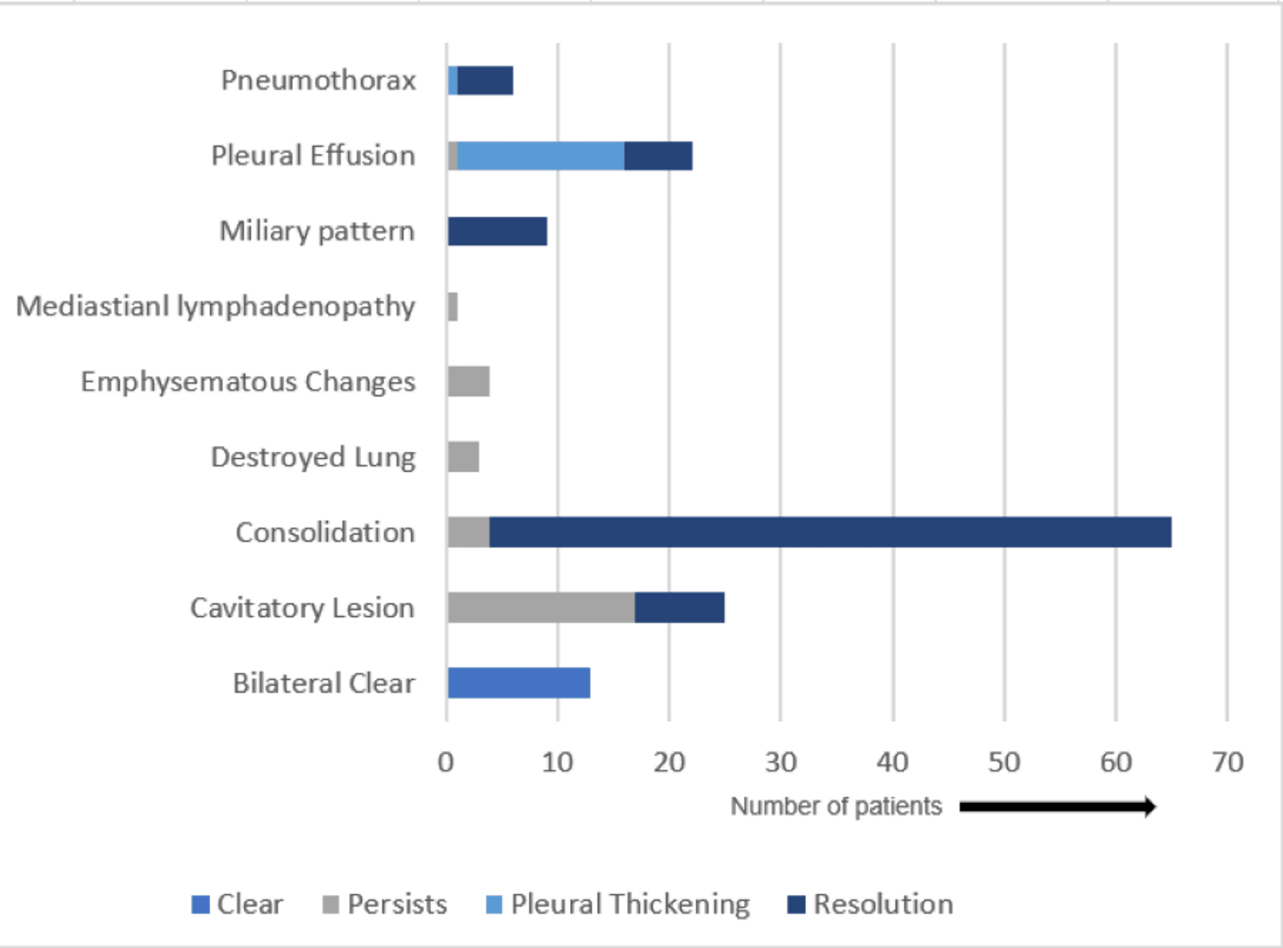
Changes in X-ray at the end of the intensive phase upon treatment with alternative regimens

A total of 60.1% (89) of the total patients showed radiographic improvement, and 93.8% (61) of consolidation cases were resolved. We subcategorized radiographic improvement into four categories: clear radiograph, resolution, pleural thickening, and persistent changes (Figure 2). No new adverse effects were observed during modified regimen use. Patients were successfully started and managed on a non-standardized regimen in accordance with the side effects shown by them. The non-standardized regimes used by us during the study were effective in managing signs and symptoms of tuberculosis.

## Discussion

A total of 141 patients were successfully assessed at the end of the intensive phase. Out of the total 148 patients enrolled in the study, 73% (108) suffered from pulmonary TB, while 27% (40) had extrapulmonary TB. More than half of the total patients, 52%(77), had tuberculosis confirmed on microbiology, while the rest, constituting 48% (71), were diagnosed on the basis of clinical signs and their symptoms on presentation. All 148 patients had their baseline investigations, including the complete blood count, liver and renal profiles, and viral markers. This helped us monitor the adverse effects of ATT by comparing deranged lab results with baseline serology. It also flagged patients with a high risk of developing adverse effects (patients with hepatitis and diabetes).

After starting standardized ATT, all patients had regular blood tests. Within the first month, 53.4% (79) of patients had abnormal liver profiles, 9.5%(14) had impaired renal function, 7.4% (11) had elevated serum uric acid titers, which required dose adjustment or drug modification in the standardized ATT therapy. Seven patients were hepatitis B or C positive before initiating treatment, with six of them developing DILI during the first month of treatment [11]. DILI (79) was diagnosed based on liver enzymes titers (aspartate aminotransferase (AST) or alanine aminotransferase (ALT)) >5 times the Upper Limit of Normal (ULN) or serum total bilirubin >2 times the ULN with symptoms. In our sample size for this study, 31.6% (25) had AST >5 times ULN, 29% (23) had ALT >5 times ULN, and 10.1% (8) had serum total bilirubin >5 mg/dL (more than twice the ULN)

Drug-induced liver injury (DILI)

Patients with DILI were started on regimens excluding pyrazinamide: E-Levo 24% (19), HRE 25% (20), SE-Levo 18% (15), HE-Levo 13% (11), and others 17% (14). In a similar study conducted by Yee et al, severe hepatitis resulted in discontinuation of H and Z in six of their patients, and neither was restarted [12]. A similar study carried out by Dhiman et al it was seen that the greatest challenge in patients with chronic liver disease or liver cirrhosis and tuberculosis is managing the therapy since the best first-line anti-tuberculosis drugs are hepatotoxic and baseline liver function is often deranged for this patient subgroup [4]. Starting these drugs can cause worsening of hepatic-related pathology, severe jaundice, vomiting, and malnutrition. None of the patients with DILI were started on pyrazinamide in our study, and many had to be managed on fluoroquinolone-based therapy (E-Levo, HE-Levo, SE-Levo).

Gastrointestinal (GI) intolerance

The regimens HRE, SE-Levo, and HE-Levo were the most common prescriptions for patients experiencing severe GI intolerance following standardized regimen initiation. Pyrazinamide was stopped in all the non-standardized regimens following GI intolerance.

Likewise, in a study conducted by Kwon et al on increased incidence of side effects seen with pyrazinamide use, it was seen that for the majority of the side effects, the causative drug was determined based on the disappearance of the effect upon drug withdrawal and/or the recurrence of the same adverse effect with rechallenge. Z was the most common drug associated with adverse effects among the first-line anti-TB drugs [13]. Pyrazinamide is usually one of the most hepatotoxic ATT first-line drugs. It causes GI intolerance and hyperuricemia, making it very difficult to be rechallenged. None of the modified regimens in our study consisted of pyrazinamide.

Renal impairment

The people with renal impairment were started on RH, RHZ, and RZ, with the addition of moxifloxacin. Patients with acute kidney injury were continued on a regimen like HRE, where the frequency of ethambutol was modified. In synchrony with the recommendations of the American Thoracic Society and others, we continued the use of nephrotoxic drug ethambutol in first-line management with dose frequency modification [8,14].

Hyperuricemia

Solangi et al, in their study on hyperuricemia induced by pyrazinamide, concluded that the change is reversible after the withdrawal of the agent. It was also noted that pyrazinamide affects serum uric acid levels very early post initiation of the standardized ATT regimen [7]. In our study, pyrazinamide was not prescribed to patients with high serum uric acid titers. They were managed effectively on HRE and HRE with levofloxacin.

Pregnancy

There was only a single case of pregnancy with tuberculosis enrolled in the study. She was managed on RHE-Levo with no pyrazinamide, although new literature suggests no contraindication to pyrazinamide use [8]. Switching to a non-standardized regimen for DILI and GI intolerance was majorly seen in adults more than 60 years of age: 23% (18) and 28% (9), respectively [15].

Out of 148 patients, 141 patients completed the intensive phase, four patients were lost to follow-up, while three died during the intensive phase (TB as a contributing cause of death) (Figure 1). Among the 141, 76% (108) patients showed improvement in symptoms, and 22% (33) had partial or no relief in symptoms like cough, breathlessness, backpain (Pott’s spine). It is seen that patients might require a longer duration of ATT due to modification of the standard regimen. The appropriate assessment would be at the end of the continuation phase, while our study only followed the patients in the intensive phase of treatment. This is one of the limitations of our study (Figure 1).

A total of 60.1% (89) of the total patients showed radiographic improvement, with 93.8% (61) of consolidation cases resolved. We subcategorized radiographic improvement into four categories: clear radiographs, resolution, pleural thickening, and persistent changes (Figure 2). No new adverse effects were observed during modified regimen use. Patients were successfully started and managed on a non-standardized regimen in accordance with the side effects shown by them. The non-standardized regimes used by us during the study were effective in managing signs and symptoms of tuberculosis.

Limitations

To highlight the significance of non-standardized ATT, a longer follow-up with a larger sample size would be required, especially until the end of the continuation phase. This long-term follow-up would also determine the difference in duration of management as compared to standardized ATT management. A follow-up study with a larger sample size comparing the efficacy of the various non-standardized regimens would be needed in the future.

Recommendations

If patients develop adverse reactions or intolerance to ATT, management with a standardized regimen should be withheld and then restarted with the least toxic drugs, trying to slowly reintroduce the standardized therapy. If not possible, treating with a minimum of three first-line drugs or with a combination of suitable alternative drugs like fluoroquinolones is advised. This study highlights the importance of the need for guidelines in the prescription of non-standardized ATT regimens. A study with a larger sample size and longer follow-up would serve as a paving stone for future research in this field.

## Conclusions

Non-standardized ATT regimens are often necessary due to adverse drug reactions of the standardized regimen, particularly hepatotoxicity. HRE-based and fluoroquinolone-containing regimens are common and generally well-tolerated alternatives. Pyrazinamide should be avoided in patients with DILI, GI intolerance, or hyperuricemia. In our study, most patients experienced symptomatic and radiological improvement, and no adverse events were observed with alternative regimens during the intensive phase. However, we strongly recommend a larger follow-up study comparing the efficacy of various non-standardized regimens noted in this study.
